# Drinking from the Data Well: Response to Gamete donor anonymity and limits on numbers of offspring: the views of three stakeholders

**DOI:** 10.1093/jlb/lsw042

**Published:** 2016-10-17

**Authors:** Martha M. Ertman

**Affiliations:** Carole & Hanan Sibel Research Professor, University of Maryland Carey Law School, Baltimore, MD 21201, USA

Data matters. Senator Cory Booker's mantra is ‘in God we trust … Everybody else, bring me data’,^1^ and the federal government's less catchy term for data-driven law and policy is ‘evidence-based practices’.^2^ Yet the privatization of family making generally—coital and technological—has translated to lack of government record-keeping, leaving scholars, journalists, courts, legislators, and advocates to shape their approaches on nightmare cases of disease and disorder or anecdotes like ads on campuses offering ivy league egg donors $30,000.^3^ Not surprisingly, these extreme stories can lead to proposals that would increase state intervention reproductive technology.

The literature review in Nelson, Hertz, and Kramer's *Gamete Donor Anonymity and Limits on Numbers of Offspring: The Views of Three Stakeholders* acknowledges existing data collection on assisted reproduction, studies that tend to offer patchwork pictures of different populations at different times and places, answering different questions. Their article represents a marked departure in its attempt to tell a broader story.

First, the data set is expansive. It compiles the views and experiences of sizeable population: 325 donors, 2134 parents, and 419 children. The researchers posed a range of questions to these donors, parents, and donor-conceived children, all at essentially the same time. Moreover, some of their respondents are in the same family, providing a detailed comparison of the three main stakeholders in assisted reproduction. As a whole, the data set should enable researchers to map similarities and differences within each stakeholder group, between stakeholder groups, and to some extent even within a particular family.

A piece of this large study appears in this volume, exploring two issues: donor anonymity and the number of offspring born of each donor. The authors provide a metric to evaluate scholarly proposals, professional practices, and legal rules regarding these two issues. The leading proponent of increased state involvement on both of these issues is Naomi Cahn, who proposes that donor registries replace anonymity and suggests stamping birth certificates ‘by donor’ for children conceived via assisted reproduction.^4^ She likewise proposes that banks and the federal government track and limit the number of children born of each donor, echoing the calls raised periodically in popular media stories about one donor providing the gametes for scores of children.^5^

Lurking behind these proposals for increased state regulation is the question of whether assisted reproduction is more like coital conception or like adoption. If it is like coital conception, then constitutional doctrines as well as privacy norms stand in the way of bans on anonymity or limits on numbers of children conceived unless the state is prepared (and constitutionally permitted) to take actions such as genetically testing all babies to make sure that the approximately 3% of babies conceived in infidelity know their genetic provenance, or mandate sterilization or birth control for particularly fecund people like the Duggans of reality television fame.^6^ More prosaically, a state office like the Center for Disease Control might produce reports that tabulate who uses alternative insemination to create families, the identity of gamete donors, how much the recipients pay for gametes, how many other children are the progeny of any given donor, and whether legal parents tell their children that they are donor conceived.^7^

Space constraints preclude a review of what Nelson, Hertz, and Kramer's data tell us about the advisability of these actions. Instead, this comment focuses on three surprising findings that should interest law makers and policy shapers who seek to conform reproductive technology rules to the lived realities of donor-conceived families. Two core principles make these three surprises stand out: family law's oft-repeated insistence on protecting children's best interests and the dangers of interfering in adults’ freedom of action and decision-making in most matters related to family creation and child raising.

## SURPRISE NO. 1: THE KIDS ARE MOSTLY ALRIGHT ABOUT ANONYMITY

As a whole, donor-conceived children in this study were not determined to abolish anonymity. Instead, just a third (33%) were ‘neutral’ about the issue of anonymity rather than for or against it.^8^ This was the most common response.

But just under a third (31%) of the offspring strongly opposed anonymity. At first glance, this datum seems to support calls for national registries and a ban on anonymous donation. Nelson, Hertz, and Kramer look closely at this anti-anonymity group, however, and find that older donor-conceived people are most likely to express hostility to anonymity. They were born decades ago, when assisted reproduction was less acceptable legally and socially, and available to married heterosexual couples more than single and gay people. Because of those norms and sometimes professional advice to not tell children about their origins, these offspring were also more likely to discover their origins relatively late in life.^9^ One woman in her 30s reported feeling as a teenager that being donor conceived was ‘a secret I was ashamed of’, and now feels ‘deeply betrayed to have been lied to, and … mad for being the object of a contract’.^10^ Similarly, a 59-year-old woman who discovered that she is donor conceived only when she was 53 years old said ‘I think anonymity should be banned.’^11^ That combination—being born to heterosexual, two-parent families and being lied to—produced a level of bitterness largely absent in younger donor-conceived offspring.

Younger respondents—in their teens and 20s—came into a world more friendly to reproductive technologies and family variety. These younger subjects were more likely to be raised by single or gay parents, and, as the authors put it, tended to emphasize that ‘families can take all different forms and that love is not based on genes’.^12^ Because single and gay parents cannot easily hide the fact of donor involvement, younger offspring also were more likely to know their origins from the outset and therefore less likely to suffer the unpleasant surprise of finding out late in childhood or as adults that the family they had was quite different from the one they thought they had. As one child born to a lesbian couple reported, ‘A family with children conceived with donated sperm is just as viable, loving, and connected as any other family. The most healthy conception of a donor for the children is for the donor to be very unimportant in their conception of their family.’^13^

Nelson, Hertz, and Kramer largely allowed the data to speak for itself. They acknowledge that the response to anonymity expressed by older donor-conceived children may be an artifact of the more homogenous social and legal conditions that shaped those offsprings’ growing up, and also the possibility that older people may be more thoughtful about the complex interplay of genetics and social relationships, especially once they have children themselves.^14^ Most relevant for purposes of evaluating proposals to ban anonymity is the authors’ conclusion that ‘the data here suggest that the debate in the USA might have overstated the extent to which donor-conceived offspring uniformly prefer openness.’^15^

## SURPRISE NO. 2: DONOR SIBLING REGISTRY (DSR) PARTICIPANTS ARE NOT ANTI-ANONYMITY

The second surprising bit of data is that offspring who have had contact with half-siblings through online networks like the DSR were more supportive of anonymity than those who have not had contact with half-siblings of the same donor (sometimes known as ‘diblings’).^16^ Since two-thirds of the offspring surveyed (267 of 419) came from the DSR database, I would expect that this population would put a premium on information about and contact with genetic kin. The authors suggest that choice may instead inform opposition to anonymity. Before DSR was created in 2000 and other online registries followed suit, donor-conceived families did not have that medium to connect, sperm banks provided less information that can be used to identify donors, and secrecy made it harder to find even basic information like the bank's designation of a donor with a particular number. Moreover, the rise of the internet has exponentially increased the ease of connection and sleuthing. In addition, the authors note, the DSR may address offspring's desire for locating and connecting with genetic kin, so that these offspring feel less need to get information from and have contact with their donors.

## SURPRISE NO. 3: LIKELY FUTURE FOCUS ON NUMBER OF OFFSPRING

Third and finally, I was surprised to read that the authors predict that future proposals for reform will focus on limiting the number of children conceived by any particular donor. This issue has been debated less in scholarly or policy circles than anonymity. The authors reason that parents and offspring report strong support for preventing one donor from siring scores of children. Notably, their data also indicate that gay and lesbian parents express less concern about this issue. They—unlike heterosexual parents—are less able to credibly lie to their children about their origins, and accidental incest is a bigger risk for those who do not know that they were donor conceived. The study also documents that gay and lesbian parents are generally more cautious about regulation, an unsurprising data point given the state's very recent transition from ignoring or punishing gay people to marriage equality and other gay rights.

I am less confident of this prediction than others put forward by Nelson, Hertz, and Kramer. Even if the issue of the number of children conceived with any particular donor matters a lot to donor-conceived families, I do not expect pushes for regulation unless partnered, heterosexual parents greatly outnumber gay parents in the overall population of donor-conceived families, and these heterosexual parents organize for lobbying efforts. Yet, the greater likelihood of this population remaining closeted about using donor gametes presents a formidable barrier to that organizing. Finally, since the fertility industry is the most organized and well-funded stakeholder in donor-conceived families, I see no reason to expect it to cede its prominence in policy and legal debates. Accordingly, while Nelson, Hertz, and Kramer provide compelling evidence that the state may increase its participation in reproductive technology arrangements, my money is on continued deregulation.^17^

## Footnotes

1 Cory Booker, *Man of Faith, Man of Data*, Atlantic Magazine event on Reinventing the War on Poverty (Mar. 17, 2014), http://www.theatlantic.com/live/articles/2014/03/cory-booker-man-data-man-faith/359242/ (last accessed August 19, 2016).

2 Edward T. Jennings, Jr. & Jeremy L. Hall, *Evidence-Based Practice and the Use of Information in State Agency Decision Making*, 22, J. Pub. Adm. Res. & Theory 245 (2012).

3 See eg Cristine Hauser, *Sperm Donor's Profile Hid Mental Illness and Crime*, NY Times, Apr. 17, 2016; Marsha Garrison, *Law Making and Baby Making*, 113 Harv. L. Rev. 835, n. 370 (2000), http://www.nytimes.com/2016/04/18/world/americas/sperm-donors-profile-hid-mental-illness-and-crime-lawsuits-say.html?_r=0 (last accessed August 19, 2016). Michael Sandel, The Case Against Perfection (2009).

4
Naomi Cahn, The New Kindship: Constructing Donor-Conceived Families 137, 142–46 (2013).

5
*Id.* at 160–62; Jacqueline Mroz, *One Sperm Donor, 150 Offspring*, NY Times, Sep. 5, 2011, http://www.gq.com/story/how-to-have-babies-sperm-donor-ed-houben (last accessed August 19, 2016); Michael Paterniti, *Stud: 106 Babies and Counting*, GQ, Oct. 1, 2015, http://www.gq.com/story/how-to-have-babies-sperm-donor-ed-houben (last accessed August 19, 2016).

6 I. Glenn Cohen, *Response: Rethinking Sperm Donor Anonymity: Of Changed Selves, Non-Identity and One Night Stands*, 100 Geo. L.J. 443, n. 58 (2011); TLC, *19 Kids and Counting*, http://www.tlc.com/tv-shows/19-kids-and-counting/ (last accessed August 19, 2016).

7 The low end of regulation would be recording of how many children are born each year via donor insemination. Rene Almeling, *The Unregulated Sperm Industry*, NY Times, Nov. 30, 2013, http://www.nytimes.com/2013/12/01/opinion/sunday/the-unregulated-sperm-industry.html?_r=0 (last accessed August 19, 2016).

8 Margaret K. Nelson, Rosanna Hertz & Wendy Kramer, *Gamete Donor Anonymity and Limits on Numbers of Offspring: The Views of Three Stakeholders*, 3 J. L. & Biosci. 1, 10 (2016).

9
*Id.* at 24.

10
*Id.* at 19.

11
*Id.* at 20.

12
*Id.* at 21.

13
*Id.*

14
*Id.* at 11.

15
*Id.* at 27.

16
*Id.* at 21.

17 For a fuller discussion of the benefits of deregulation and dangers of government meddling in reproductive technologies, see Martha M. Ertman, Love's Promises: How Formal & Informal Contracts Shape All Kinds of Families (2015).

